# 

**Published:** 1886-06

**Authors:** 


### CHICAGO AS A MEDICAL CENTER.

On the fifteenth of June, the first session of the POST GRADUATE MEDICAL SCHOOL OF CHICAGO will commence. The CHICAGO POLYCLINIC will begin its courses of instruction for practitioners of medicine early in July. The establishment of these two schools marks a significant advance in the importance of Chicago as a medical center.

The large amount of clinical and pathological material placed at the command of these institutions will enable the practitioner to pursue his studies with ease and profit.

### BOOKS RECEIVED.

*Electricity in Medicine.* By Ambrose L. Ranney, M. D. New York: William Wood & Co. Chicago: W. T. Keener.

*A Manual of Operative Surgery.* By Lewis A. Stimson, B. A., M. D. Second edition. Philadelphia: Lea Brothers & Co. Chicago: A. C. McClurg & Co.

*The Blot Upon the Brain.* By William W. Ireland, M. D., Edin. New York: G. P. Putnam's Sons. Chicago: A. C. McClurg & Co.

*The Principles and Practice of Medicine.* By Charles Hilton Fagge, M. D., F. R. C. P. Volume I. Philadelphia: P. Blakiston, Son & Co. Chicago: W. T. Keener.

*The Science and Art of Surgery.* By John Eric Erichsen, F. R. S., LL.D., F. R. C. S. Philadelphia: Lea Brothers & Co. Chicago: A. C. McClurg & Co.

*The Diseases of Sendentary and Advanced Life.* By J. Milner Fothergill, M. D. New York: D. Appleton & Co. Chicago: A. C. McClurg & Co.

*Transactions of the American Surgical Association.* Volume III. Philadelphia: P. Blakiston, Son & Co. Chicago: W. T. Keener.

*An Atlas of Clinical Microscopy.* By Alexander Peyer, M. D.

*The Principles and Practice of Surgery.* By John Ashurst, Jr., M. D. Fourth edition. Philadelphia: Lea Brothers & Co. Chicago: A. C. McClurg & Co.

*Lectures on the Principles of House Drainage.* By J. Pickering Putnam. Boston: Ticknor & Co. Chicago: A. C. McClurg & Co.

*A Manual of Microscopical Technology.* By Stephen Yates Howell, A. M., M. D. New York: G. P. Putnam's Sons. Chicago: A. C. McClurg & Co.

*Essentials of Vaccination.* By W. A. Hardaway, M. D. St. Louis: J. H. Chambers & Co.

*Insanity and its Treatment.* By G. Fielding Blandford, M. D., Oxon. New York: William Wood & Co. Chicago: W. T. Keener.

*Diseases of the Spinal Cord.* By Byrom Bramwell, M. D., F. R. C. P., (Edin.). New York: William Wood & Co. Chicago: W. T. Keener.

*A Compend of Pharmacy.* By F. E. Stewart, M. D., Ph. G. Philadelphia: P. Blakiston Son & Co. Chicago: W. T. Keener.

*Saint Bartholomew's Hospital Reports.* Volume XXI. Edited by W. S. Church, M. D., and John Langton, F. R. C. S. London: Smith, Elder & Co.

*Twelfth Annual Report* of the Secretary of the State Board of Health, of the State of Michigan.

*The Principles and Practice of Surgery.* By Frank Hastings Hamilton, A. M., M. D., LL.D. New York: William Wood & Co. Chicago: W. T. Keener.

*A Treatise on Bright's Disease of the Kidneys.* By Henry B. Millard, M. D., A. M. New York: William Wood & Co. Chicago: W. T. Keener.

*A Reference Handbook of the Medical Sciences.* Volume II. New York: William Wood & Co. Chicago: W. T. Keener.

*The International Encyclopædia of Surgery.* Volume VI. New York: William Wood & Co. Chicago: W. T. Keener.

*Dictionary of Practical Surgery.* Edited by Christopher Heath, F. R. C. S. Philadelphia: J. B. Lippincott & Co. Chicago: A. C. McClurg & Co.

*Handbook of Practical Medicine.* By Dr. Hermann Eichorst. New York: William Wood & Co. Chicago: W. T. Keener.

*A Manual of the Diseases of the Skin.* By Balmano Squire, M. D., London. Chicago: A. N. Marquis & Co.

*Lectures on Dietetics and Dyspepsia.* By Sir William Roberts, M. D., F. R. S. London: Smith, Elder & Co. Chicago: A. C. McClurg & Co.

*Haschisch.* By Thorold King. Chicago: A. C. McClurg & Co.

*A Manual of Surgery.* Volume I. Philadelphia: Lea Brothers & Co. Chicago: A. C. McClurg & Co.

*Surgical Diseases of the Kidneys.* By Henry Morris, M. A., M. B., F. R. C. S. Philadelphia: Lea Brothers & Co. Chicago: A. C. McClurg & Co.

*A Manual of Surgery.* Volume II. Philadelphia: Lea Brothers & Co. Chicago: A. C. McClurg & Co.

*The Student's Manual of Venereal Diseases.* By Berkeley Hill, M. D., and Arthur Cooper, M. D. Philadelphia: P. Blakiston, Son & Co. Chicago: W. T. Keener.

*A Manual of Surgery.* Volume III. Philadelphia: Lea Brothers & Co. Chicago: A. C. McClurg & Co.

*The Surgical Diseases of Children.* By Edmund Owen, M. B., F. R. C S. Philadelphia: Lea Brothers & Co. Chicago: A. C. McClurg & Co.

*The Genuine Works of Hippocrates.* By Francis Adams, LL.D. Volume I. New York: William Wood & Co. Chicago: W. T. Keener.

*Diseases of the Digestive Organs in Infancy and Childhood.* By Louis Starr, M. D. Philadelphia: P. Blakiston, Son & Co. Chicago: W. T. Keener.

*First Annual Report of the State Board of Health of the State of Kansas.*

*The Best Preliminary Education for the Study of Medicine.* Boylston Prize Essay, 1885.

*Drainage for Health, or Easy Lessons in Sanitary Science.* By Joseph Wilson, M. D. Philadelphia: P. Blakiston, Son & Co. Chicago: W. T. Keener.

### INDEX TO VOLUME LII.

A

Page.

"A Complicated Labour," Skeen ........ 199
Acne, Review ........ 86
Albuminuria, Acute, Hall ........ 201
American Academy of Medicine 39
American Laryngological Association ........ 567
American Medical Association. ........ 325, 383, 564
American Public Health Association, Editorial ........ 14
American Surgical Association. 567
Andrews, E., Surgical Clinic. 69
Antipyrin in Typhoid Fever, Mannheimer ........ 97
Antiseptics, Notes on New, Levis ........ 239
Association of American Medical Editors ........ 567
Axford, W. L., Removal of Lower Jaw ........ 479
Axis-Traction Forceps, Jaggard 153

B

Babcock, Robert H., Mitral Stenosis ........ 475
Bacillus Tuberculosis, Schmidt. ........ 393, 489
Bacteria Investigation, Technology of, Review ........ 81
Bacteria, Relation of to Puerperal Inflammations, Cushing ........ 44
Belfield, W. T., Exploration of the Bladder ........ 363
Belfield, W. T., Stricture of the Urethra ........ 554
Bladder, Digital Exploration of, Belfield ........ 363

Page

Books Received ........ 91, 391, 575
Brower, D. R., Cocaine on the Nervous System ........ 173
Byford, H. T., Laceration of the Perineum ........ 371
Byford, Henry T., Pelvic Abscess ........ 7, 160

C

Chicago Gynæcological Society.
Meeting Nov. 27, 1885 ........ 24
" Dec. 18, 1885 ........ 153
" Jan. 15, 1886 ........ 268
" Feb. 19, 1886 ........ 330
" Mar. 19, 1886 ........ 536
Chicago Medical College ........ 378
Chicago Medical Society.
Meeting Dec. 21, 1885 ........ 19
" Dec. 7, 1885 ........ 21
" Jan. 4, 1886 ........ 173
" Jan. 18, 1886 ........ 257
" Feb. 1, 1886 ........ 352
" Feb. 15, 1886 ........ 363
" Mar. 1, 1886, ........ 463
" Mar. 15, 1886 ........ 475
" April 19, 1886 ........ 553
Chicago Society of Opthalmology and Otology.
Meeting Oct. 13, 1885 ........ 29
Chicago Water Supply ........ 94
Chinese-American Doctors ........ 95
Chloral Hydrate in Emesis, Marr ........ 238
Cholecystotomy, Keen ........ 249
Choroid, Ossification of, Ware ........ 29
Choroid, Ossification of, Coleman ........ 376
Choroiditis following Typhoid Fever, Hotz ........ 33

PAGE.

Climatic Treatment of Disease, Marcy ... 40
Climatology and Mineral Waters of the United States, Review ... 386
Cocaine, Effects of on the Central Nervous System, Brower 173
Cocaine in Hay-fever, Da Costa 35
Coleman, W. F., Ossification of the Choroid ... 376
College of Physicians and Surgeons of Chicago ... 378
College of Physicians of Philadelphia.
Meeting Oct. 7, 1885 ... 35
" Nov. 4, 1885 ... 56
Cook County Hospital ... 379
Cook County Insane Asylum, Editorial ... 233
Consumption, Review ... 188

D

Da Costa, J. M., Cocaine in Hay-fever ... 35
Didama, Henry, Tubercular Consumption ... 46
Digestion and Dyspepsia, Jensen ... 463
Doering, E. J., Hydatiform Pregnancy ... 112, 279
Doering, E. J., Protection Against Blackmail ... 257
Dudley, E. C., Vesico-Vaginal Fistula ... 476

E

Earle, Charles Warrington, ... 104, 138, 268
Electricity in Medicine, Review. 389
Electrolysis in Stricture of the Urethra ... 554
Epilepsy, Review ... 385
Epithelioma of the Uterus, Miller ... 551
Essentials of Physics and Chemistry, Review ... 387
Etheridge, James H., Fibrous Tumor of the Uterus ... 1
Etheridge, J. H., Vesico-Vaginal Fistula ... 464

F

Feeding-Bottle, Waxham ... 351
Fenger, C., Osteo-Plastic Resection ... 560

PAGE.

Fibrous Tumor of the Uterus, Etheridge ... 1
Discussion ... 19
Fistula of the Neck, Andrews ... 69
Forceps, Obstetric ... 536
Frank, J., Degenerated Kidney ... 473

G

Gihon, Albert L., Address ... 40
Gilmore, Arnold P., Symblepharon ... 480
Gowers, W. R., Review ... 573
Grant, W. W., Pelvic Hæmatocele ... 318
Gray, John P., State License of Medical Practitioners ... 45
Gross, F. H., Suture in Deep Wounds ... 245

H

Hall, C. H. H., Acute Albuminuria ... 201
Hay-fever, Cocaine in, Da Costa. 35
Horwitz, Orville, Sunstroke ... 36
Hotz, F. C., Choroiditis ... 33
House Drainage, Review ... 385
"How Does a Doctor Look to a Clergyman?" ... 95
Human Osteology, Review ... 83
Hydatiform Pregnancy, Doering ... 112, 279
Hyde, James Nevins, Skin Affections ... 116
Hygienic Clothing, McArthur ... 144
Histology, Essentials of, Review ... 486

I

Illinois State Board of Health. Meeting, 29th Oct., '85 ... 77
Illinois State Medical Society ... 236, 563
Indiana State Board of Health ... 79
Inorganic Chemistry, Review ... 388
Insane Asylums, Female Physicians in, Paoli ... 553
Insane Hospital Supervision, Spitzka ... 289
Insanity from Cataract Operations ... 382
International Medical Congress ... 15, 94, 568

PAGE.

Intubation of the Larynx, Waxham ... 214
Intubing the Larynx, Strong ... 193, 353
"Ipsis Consuescere Rebus," Editorial ... 462

J

Jackson, A. Reeves, Discussion. 539
Jaggard, W. W., Axis-Traction Forceps ... 153
Jensen, P. C., Digestion and Dyspepsia ... 463

K

Keen, Wm. W., Cholecystotomy ... 248
Keen, W. W., Tait's Operation ... 56
Knox, J. Suydam, Quinsy ... 208, 352

L

Labour and the Lying-in Period, Review ... 483
Lagorio, A., Letter ... 329
Lanolin, Tilley ... 474
Laws Regulating the Practice of Medicine, Report ... 43
Levis, R. J., New Antiseptics ... 239
Lupus of the Larynx, Stockton ... 74
Lupus Vulgaris, Reynolds ... 64
Lymphadenoma, Case of, Nelson ... 525

M

Mann, Edward C., Paralysis Agitans ... 517
Mannheimer, M., Antipyrin ... 97
Marcy, Henry O., Climatic Treatment of Disease ... 40
Marr, D. D., Letter ...
McArthur, L. L., Hygienic Clothing ... 144
McIntyre, C., Student Life ... 40
Medical Evidence, Turner ... 42
Medicine, Study of, as a Means of Education, Sibbett ... 39
Michigan State Board of Health 80
Miller, DeLaskie, Epithelioma of the Uterus ... 551
Mitral Stenosis, Babcock ... 475
Mutual Protection Against Blackmail, Doering ... 257

PAGE.

N

Nelson, J. W., Lymphadenoma 525
Neurological Review ... 381
New York State Medical Association ... 45
Nursing, Text-Book of, Review 82

O

Obstetrics, Theory and Practice of, Review ... 485
Opium-Smoking in Chicago, Earle ... 104
Ossification of the Choroid, Ware ... 29

P

Pamphlets Received ... 93, 392
Paoli, G. C., Female Physicians ... 553
Paralysis Agitans, Mann ... 517
Parkes, C. T., Sarcoma of Thigh. 482
Patella, Fractures of, Staples ... 438
Pedigree of Disease, Review ... 85
Perineum, Laceration of the, Byford ... 371
Pelvic Abscess, Byford ... 7, 160
Pelvic Abscess, Laparotomy for, Discussion ... 330
Pelvic Cellulitis, Discussion ... 539
Pelvic Hæmatocele, Grant ... 318
Philadelphia Academy of Surgery ... 239
Physicians' Protective Association, Editorial ... 460
Pneumonia, Discussion ... 47
Pneumonic Abscess, Wells ... 21
Poisons, Case of Antidotes for, Webster ... 472

Q

Quinsy as a Rheumatism, Knox. ... 208, 352

R

Rationalism in Medical Treatment, Review ... 84
Reynolds, Henry J., Clinical Lecture ... 64
Rush Medical College ... 378

PAGE.

S

Sanitary Convention ... 191
Sarcoma of Thigh, Parkes ... 482
Schmidt, H. D., Bacillus Tuberculosis ... 389, 493
Sibbett, R. L., Study of Medicine ... 39
Skeer, John D., A Complicated Labour ... 199
Skin Affections in Cold Weather, Hyde ... 116
Spiritism, Review ... 287
Spitzka, E. C., Insane Hospital Supervision ... 289
Staples, George A., Inaugural Thesis ... 438
State License of Medical Practitioners, Gray ... 45
State Medicine, Carroll ... 46
Stockton, Frank O., Clinical Lecture ... 74
Stone, Alex. J., Forceps ... 536
St. Paul Medical School ... 381
Strong, Albert B., Intubing the Larynx ... 193, 353
Student Life, Medical Supervision of, McIntyre ... 40
Sunstroke, Treatment of, Horwitz ... 36
Surgery, A Manual for the Practice of, Review ... 387
Symblepharon, Gilmore ... 480
Synovitis of Knee, Andrews ... 71

PAGE.

T

Tait's Operation, Keen ... 56
Tilley, R., Lanolin ... 474
Tubercular Consumption, Didama ... 46
Turner, T. J., Medical Evidence. 42
Tyson, James, Review ... 574

U

Urinary and Renal Diseases, Review ... 189

V

Valin, H. D., Sex in Utero ... 265
Vesico-Vaginal Fistula, Dudley. 476
Vesico-Vaginal Fistula, Etheridge ... 464

W

Ware, Lyman, Ossification of the Choroid ... 29
Watery Discharges of Pregnant Women, Earle ... 138, 268
Waxham, F. E., Feeding Bottle ... 351
Waxham, F. E., Intubation of the Larynx ... 214
Webster, G. W., Case of Poison Antidotes ... 472
Wells, Edward F., Pneumonic Abscess ... 21
"What is Medicine?" An Address, Gihon ... 40

SUBSCRIPTION PRICE, $3.00 PER YEAR.

hole Number, 317. JUNE, 1886. Vol. LII., Number 6.

THE

### CHICAGO MEDICAL JOURNAL AND EXAMINER

EDITORS:

S. J. JONES, M.D., LL.D.

W. W. JAGGARD, A.M., M.D. HAROLD N. MOYER, M.D.

COLLABORATORS.

CHICAGO:

PROFESSOR J. A. ALLEN, M. D., LL. D.

PROFESSOR E. ANDREWS, M. D., LL. D.

PROFESSOR W. H. BYFORD, A. M., M. D.

PROFESSOR O. C. DEWOLF, A. M., M. D.

PROFESSOR E. C. DUDLEY, A. B., M. D.

PROFESSOR J. H. ETHERIDGE, A. M., M. D.

PROFESSOR C. FENGER, M. D.

PROFESSOR J. N. HYDE, A. M., M. D

PROFESSOR E. F. INGALS, A. M., M.D.

PROFESSOR H. A. JOHNSON, M. D., LL. D.

CHARLES GILMAN SMITH, A. M., M.D,

J. F. TODD, M. D.

MILWAUKEE, WISCONSIN:

PROFESSOR N. SENN, M. D.

WASHINGTON, D. C:

S. O. RICHEY, M. D.

UNITED STATES ARMY:

SURGEON, D. L. HUNTINGTON, M. D.

UNITED STATES NAVY:

MEDICAL DIRECTOR, J. M. BROWNE, M. D

MEDICAL DIRECTOR, A. L. GIHON, A. M., M. D.

